# Thorium divanadate dihydrate, Th(V_2_O_7_)(H_2_O)_2_

**DOI:** 10.1107/S1600536811039584

**Published:** 2011-09-30

**Authors:** Said Yagoubi, Lahcen El Ammari, Abderrazzak Assani, Mohamed Saadi

**Affiliations:** a Groupe de Radiochimie, Institut de Physique Nucléaire d’Orsay UMR 8608, Université de Paris-Sud-11, Bât. 100, 91406 Orsay, France; b Laboratoire de Chimie du Solide Appliquée, Faculté des Sciences, Université Mohammed V-Agdal, Avenue Ibn Battouta, BP 1014, Rabat, Morocco

## Abstract

The title compound, Th(V_2_O_7_)(H_2_O)_2_, was synthesized by a hydro­thermal reaction. The crystal structure consists of ThO_7_(OH_2_)_2_ tricapped trigonal prisms that share edges, forming [ThO_5_(OH_2_)_2_]_*n*_ chains along [010]. The edge-sharing ThO_7_(OH_2_)_2_ polyhedra share one edge and five vertices with the V_2_O_7_ divanadate anions having a nearly ecliptic conformation parallel to [001]. This results in an open framework with the water mol­ecules located in channels. O—H⋯O hydrogen bonding between water molecules and framework O atoms is observed. Bond-valence-sum calculations are in good agreement with the chemical formula of the title compound.

## Related literature

For thorium compounds with ninefold coordination of the metal, see: Matkovic *et al.* (1968 ▶); Boatner (2002 ▶); Sullens & Albrecht-Schmitt (2005 ▶); Sullens *et al.* (2006 ▶); Calestani & Andreetti (1984 ▶); Kojić-Prodić *et al.* (1982 ▶). For bond-valence sums, see: Brese & O’Keeffe (1991 ▶).

## Experimental

### 

#### Crystal data

Th(V_2_O_7_)(H_2_O)_2_
*M* *_r_* = 481.95
Triclinic, 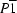

*a* = 7.0432 (4) Å
*b* = 7.3702 (4) Å
*c* = 7.7204 (4) Å
α = 77.849 (2)°
β = 74.831 (2)°
γ = 85.934 (2)°
*V* = 378.08 (4) Å^3^
*Z* = 2
Mo *K*α radiation
μ = 22.06 mm^−1^
*T* = 296 K
0.19 × 0.12 × 0.10 mm

#### Data collection

Bruker X8 APEXII diffractometer
Absorption correction: multi-scan (*SADABS*; Bruker, 2005 ▶) *T*_min_ = 0.052, *T*_max_ = 0.110
15216 measured reflections
3576 independent reflections
3506 reflections with *I* > 2σ(*I*)
*R*_int_ = 0.031

#### Refinement

*R*[*F* ^2^ > 2σ(*F* ^2^)] = 0.014
*wR*(*F* ^2^) = 0.033
*S* = 1.15
3576 reflections
110 parameters
H-atom parameters constrained
Δρ_max_ = 1.60 e Å^−3^
Δρ_min_ = −1.26 e Å^−3^

### 

Data collection: *APEX2* (Bruker, 2005 ▶); cell refinement: *SAINT* (Bruker, 2005 ▶); data reduction: *SAINT*; program(s) used to solve structure: *SHELXS97* (Sheldrick, 2008 ▶); program(s) used to refine structure: *SHELXL97* (Sheldrick, 2008 ▶); molecular graphics: *ORTEP-3 for Windows* (Farrugia, 1997 ▶) and *DIAMOND* (Brandenburg, 2006 ▶); software used to prepare material for publication: *WinGX* (Farrugia, 1999 ▶).

## Supplementary Material

Crystal structure: contains datablock(s) I, global. DOI: 10.1107/S1600536811039584/fj2454sup1.cif

Structure factors: contains datablock(s) I. DOI: 10.1107/S1600536811039584/fj2454Isup2.hkl

Additional supplementary materials:  crystallographic information; 3D view; checkCIF report

## Figures and Tables

**Table 1:** Hydrogen-bond geometry (Å, °)

*D*—H⋯*A*	*D*—H	H⋯*A*	*D*⋯*A*	*D*—H⋯*A*
O8—H8*A*⋯O4^i^	0.86	2.47	3.114 (3)	133
O8—H8*B*⋯O5^ii^	0.86	1.92	2.779 (3)	175
O9—H9*A*⋯O1^iii^	0.86	1.77	2.594 (3)	160
O9—H9*B*⋯O5^iv^	0.86	1.90	2.741 (3)	166

Symmetry codes: (i) 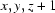; (ii) 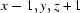; (iii) 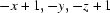; (iv) 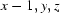.

**Fig. 1.**
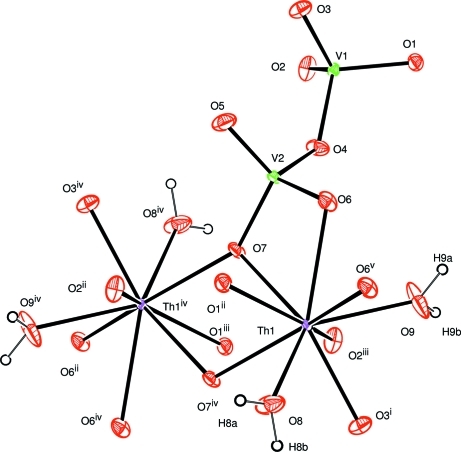
Partial plot of Th(V2O7)(H2O)2 crystal structure. Displacement ellipsoids are drawn at the 50% probability level. Symmetry codes: (i) x - 1, y, z + 1; (ii) x, y, z + 1; (iii) -x + 1, -y + 1, -z + 1; (iv) -x + 1, -y + 1, -z + 2; (v) -x + 1, -y, -z + 2; (vi) -x + 1, -y, -z + 1; (vii) x - 1, y, z.

**Fig. 2.**
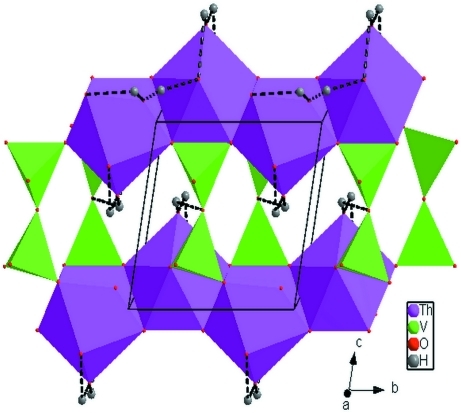
A three-dimensional polyhedral view of the crystal structure of the Th(V2O7)(H2O)2 showing polyhedra linkage.

## supplementary crystallographic information

### Comment

As reported in the literature, a few structures with ninefold coordination of thorium have been observed. In the case of orthophosphates and orthoarsenates, only two structure-types exist: the monoclinic KTh_2_(PO_4_)_3_ (space group *C*2/*c*) (Matkovic *et al.* 1968) and the monazite CePO_4_ (space group P21/n) structures (Boatner, 2002). The KTh_2_(PO_4_)_3_ structure is built from corrugated sheets parallel to (100) face of thorium polyhedra ThO_9_ sharing edges. Phosphate tetrahedral share vertices and edges with the thorium polyhedra to define a framework with channels occupied by K atoms. For the monazite compound, the structure shows a parallel chains to [010] direction, formed by CeO_9_ polyhedra that share edges. These infinite chains are connected together by edge-sharing with phosphate tetrahedra to form sheets parallel to the (100) face. The stacking of these layers along [100] direction by further edge-sharing of the CeO_9_ polyhedra forms a three-dimensional framework. These two structure-types (KTh_2_(PO_4_)_3_ and CePO_4_) are amongst the most important as they show the widest range of chemical composition. Dimers of edges-shared ThO_9_ are found in Na_6_[Th(PO_4_)(P_2_O_7_)]_2_ (space group P-1) (Kojić-Prodić, *et al.*, 1982). These dimmers are connected together by sharing edges with phosphate tetrahedral into double chains along [100] direction. Pyrophosphate groups share vertices with the double chains and define a framework with channels occupied by Na atoms. Five other Th(IV) compounds with ninefold coordination and heavy oxoanions were cited in the literature: four structures containing mixed geometry anions were published by Sullens & Albrecht-Schmitt (2005); Sullens *et al.* (2006), Th(VO_2_)_2_(TeO_6_)(H_2_O)_2_, Th(SeO_3_)(SeO_4_), Th(IO_3_)_2_(SeO_4_)(H_2_O)_3_ H_2_O and Th(CrO_4_)(IO_3_)_2_ and one compound with mixed site Pb_0.5_Th_0.5_VO_4_ was published by Calestani & Andreetti (1984). For all these compounds, each ThO_9_ (or ThPbO_9_) polyhedra is bounded by the oxoanions groups.

In an effort to understand the structural chemistry of vanadate with actinides, we obtained the following compound of formula Th(V_2_O_7_)(H_2_O)_2_ under mild hydrothermal conditions. The structure of this compound consists of ThO_7_(OH_2_)_2_ tricapped trigonal prisms that share edges to form chains [ThO_5_(OH_2_)_2_]∞ along the [010] direction. The edge-sharing ThO_7_(OH_2_)_2_ polyhedra share one edge and five vertices with the V_2_O_7_ divanadate anions parallel to [001] direction. That builds an open framework with water molecules pointing towards the tunnels. The bond-valence sums were calculated using the coordination-independent parameters given by Brese and O'Keeffe (1991). The obtained value are as follows: Th1, 4.06; V1, 5.10 and V2, 5.18. The two oxygen atoms (O8 and O9) are concluded to be water molecules on the basis of their high isotropic displacement parameters, their bond valence sums of 0.34 (O8) and 0.47(O9), charge balance requirements. That gives the structural formula Th(V_2_O_7_)(H_2_O)_2_ for the studied compound.

### Experimental

Crystals of Th(V_2_O_7_)(H_2_O)_2_, were hydrothermally synthesized in a 25 ml Teflon-lined steel autoclave from two mixtures: Th(NO_3_)_4_(H_2_O)_4_, NH_4_VO_3_ and V_2_O_5_ in the equimolar ratio or Th(NO_3_)_4_(H_2_O)_4_ and V_2_O_5_ in the molar ratio 4:3. Ten ml of distilled water was added in each mixture with pH = 4.5. The autoclaves were heated 7 days at 403 K then 2 days at 483 K followed by slow cooling to room temperature. The resulting product was recovered by filtration, washed with deionized water and finally air dried. The reaction product consists of orange powder and some yellow-colored crystals corresponding to the title compound which they can be isolated using ultrasonic.

### Refinement

The O-bound H atoms were initially located in a difference map and refined with O—H distance restraints of 0.86 (1). In a the last cycle they were refined in the riding model approximation with *U*_iso_(H) set to 1.2*U*_eq_(O). The highest and deepest hole residual peak in the final difference Fourier map are located at 0.67 Å and 0.88 Å, from Th1. The non significant distances and angles are removed from the cif file.

### Crystal data

**Table:**

Th(V_2_O_7_)(H_2_O)_2_	*Z* = 2
*M**_r_* = 481.95	*F*(000) = 424
Triclinic, *P*1	*D*_x_ = 4.233 Mg m^−^^3^
Hall symbol: -P 1	Mo *K*α radiation, λ = 0.71073 Å
*a* = 7.0432 (4) Å	Cell parameters from 3576 reflections
*b* = 7.3702 (4) Å	θ = 2.8–36.0°
*c* = 7.7204 (4) Å	µ = 22.06 mm^−^^1^
α = 77.849 (2)°	*T* = 296 K
β = 74.831 (2)°	Prism, yellow
γ = 85.934 (2)°	0.19 × 0.12 × 0.10 mm
*V* = 378.08 (4) Å^3^	

### Data collection

**Table:**

Bruker X8 APEXII diffractometer	3576 independent reflections
Radiation source: fine-focus sealed tube	3506 reflections with *I* > 2σ(*I*)
graphite	*R*_int_ = 0.031
φ and ω scans	θ_max_ = 36.0°, θ_min_ = 2.8°
Absorption correction: multi-scan (*SADABS*; Bruker, 2005)	*h* = −11→11
*T*_min_ = 0.052, *T*_max_ = 0.110	*k* = −12→12
15216 measured reflections	*l* = −12→12

### Refinement

**Table:**

Refinement on *F*^2^	Secondary atom site location: difference Fourier map
Least-squares matrix: full	Hydrogen site location: difference Fourier map
*R*[*F*^2^ > 2σ(*F*^2^)] = 0.014	H-atom parameters constrained
*wR*(*F*^2^) = 0.033	*w* = 1/[σ^2^(*F*_o_^2^) + (0.0019*P*)^2^ + 0.3878*P*] where *P* = (*F*_o_^2^ + 2*F*_c_^2^)/3
*S* = 1.15	(Δ/σ)_max_ = 0.003
3576 reflections	Δρ_max_ = 1.60 e Å^−^^3^
110 parameters	Δρ_min_ = −1.26 e Å^−^^3^
0 restraints	Extinction correction: *SHELXL97* (Sheldrick, 2008), Fc^*^=kFc[1+0.001xFc^2^λ^3^/sin(2θ)]^-1/4^
Primary atom site location: structure-invariant direct methods	Extinction coefficient: 0.0083 (3)

### Special details

**Table:**

Geometry. All e.s.d.'s (except the e.s.d. in the dihedral angle between two l.s. planes) are estimated using the full covariance matrix. The cell e.s.d.'s are taken into account individually in the estimation of e.s.d.'s in distances, angles and torsion angles; correlations between e.s.d.'s in cell parameters are only used when they are defined by crystal symmetry. An approximate (isotropic) treatment of cell e.s.d.'s is used for estimating e.s.d.'s involving l.s. planes.
Refinement. Refinement of *F*^2^ against all reflections. The weighted *R*-factor *wR* and goodness of fit *S* are based on *F*^2^, conventional *R*-factors *R* are based on *F*, with *F* set to zero for negative *F*^2^. The threshold expression of *F*^2^ > σ(*F*^2^) is used only for calculating *R*-factors(gt) *etc*. and is not relevant to the choice of reflections for refinement. *R*-factors based on *F*^2^ are statistically about twice as large as those based on *F*, and *R*- factors based on all data will be even larger.

### Fractional atomic coordinates and isotropic or equivalent isotropic displacement parameters (Å^2^)

**Table:**

	*x*	*y*	*z*	*U*_iso_*/*U*_eq_	
Th1	0.358260 (9)	0.244262 (9)	1.084559 (8)	0.00611 (3)	
V1	0.79469 (5)	0.33646 (5)	0.25516 (4)	0.00702 (6)	
V2	0.72488 (5)	0.29521 (5)	0.70555 (4)	0.00705 (6)	
O7	0.6164 (2)	0.4536 (2)	0.8411 (2)	0.0112 (3)	
O1	0.6561 (2)	0.2001 (2)	0.1850 (2)	0.0122 (3)	
O6	0.6084 (3)	0.0958 (2)	0.8397 (2)	0.0137 (3)	
O3	1.0237 (2)	0.2469 (2)	0.2411 (2)	0.0153 (3)	
O2	0.7983 (3)	0.5430 (2)	0.1207 (2)	0.0162 (3)	
O4	0.6682 (3)	0.3462 (2)	0.4897 (2)	0.0151 (3)	
O5	0.9622 (3)	0.2782 (3)	0.6782 (2)	0.0191 (3)	
O8	0.2995 (3)	0.1801 (3)	1.4332 (2)	0.0250 (4)	
H8A	0.3836	0.1644	1.4984	0.030*	
H8B	0.1908	0.2063	1.5061	0.030*	
O9	0.1870 (4)	0.1056 (3)	0.9082 (4)	0.0366 (6)	
H9A	0.2211	0.0097	0.8594	0.044*	
H9B	0.1036	0.1642	0.8519	0.044*	

### Atomic displacement parameters (Å^2^)

**Table:**

	*U*^11^	*U*^22^	*U*^33^	*U*^12^	*U*^13^	*U*^23^
Th1	0.00668 (4)	0.00456 (3)	0.00738 (3)	0.00008 (2)	−0.00182 (2)	−0.00181 (2)
V1	0.00677 (13)	0.00767 (13)	0.00671 (12)	−0.00029 (10)	−0.00215 (10)	−0.00106 (10)
V2	0.00842 (13)	0.00610 (13)	0.00644 (12)	0.00028 (10)	−0.00092 (10)	−0.00219 (10)
O7	0.0146 (7)	0.0082 (6)	0.0112 (6)	0.0006 (5)	−0.0021 (5)	−0.0050 (5)
O1	0.0109 (6)	0.0117 (7)	0.0160 (6)	0.0013 (5)	−0.0050 (5)	−0.0054 (5)
O6	0.0204 (8)	0.0070 (6)	0.0129 (6)	−0.0018 (6)	−0.0026 (6)	−0.0016 (5)
O3	0.0097 (6)	0.0200 (8)	0.0139 (6)	0.0018 (6)	−0.0020 (5)	−0.0006 (6)
O2	0.0186 (8)	0.0122 (7)	0.0176 (7)	−0.0045 (6)	−0.0092 (6)	0.0049 (6)
O4	0.0159 (7)	0.0211 (8)	0.0084 (6)	0.0043 (6)	−0.0033 (5)	−0.0046 (5)
O5	0.0100 (7)	0.0303 (10)	0.0163 (7)	0.0025 (7)	−0.0016 (6)	−0.0063 (7)
O8	0.0151 (8)	0.0467 (13)	0.0123 (7)	0.0038 (8)	−0.0024 (6)	−0.0066 (7)
O9	0.0460 (13)	0.0252 (10)	0.0645 (15)	0.0236 (10)	−0.0464 (13)	−0.0327 (11)

### Geometric parameters (Å, °)

**Table:**

Th1—O3^i^	2.3508 (16)	V1—O1	1.7037 (16)
Th1—O1^ii^	2.3988 (15)	V1—O4	1.8109 (16)
Th1—O2^iii^	2.4104 (15)	V2—O5	1.6285 (17)
Th1—O7^iv^	2.4430 (16)	V2—O6	1.7296 (16)
Th1—O9	2.4440 (19)	V2—O7	1.7351 (16)
Th1—O6^v^	2.4598 (16)	V2—O4	1.7722 (15)
Th1—O8	2.5593 (17)	O8—H8A	0.8599
Th1—O7	2.5706 (16)	O8—H8B	0.8599
Th1—O6	2.5937 (17)	O9—H9A	0.8600
V1—O2	1.6498 (16)	O9—H9B	0.8600
V1—O3	1.6845 (16)		
			
O3^i^—Th1—O1^ii^	133.04 (5)	O6^v^—Th1—O7	123.52 (5)
O3^i^—Th1—O2^iii^	74.96 (6)	O8—Th1—O7	128.58 (6)
O1^ii^—Th1—O2^iii^	141.47 (6)	O3^i^—Th1—O6	142.46 (6)
O3^i^—Th1—O7^iv^	87.85 (6)	O1^ii^—Th1—O6	74.59 (5)
O1^ii^—Th1—O7^iv^	79.21 (5)	O2^iii^—Th1—O6	97.58 (6)
O2^iii^—Th1—O7^iv^	75.88 (6)	O7^iv^—Th1—O6	126.68 (5)
O3^i^—Th1—O9	74.46 (8)	O9—Th1—O6	69.48 (7)
O1^ii^—Th1—O9	139.27 (6)	O6^v^—Th1—O6	64.61 (6)
O2^iii^—Th1—O9	63.63 (7)	O8—Th1—O6	130.52 (6)
O7^iv^—Th1—O9	138.65 (6)	O7—Th1—O6	61.59 (5)
O3^i^—Th1—O6^v^	93.95 (6)	O2—V1—O3	111.26 (9)
O1^ii^—Th1—O6^v^	77.24 (6)	O2—V1—O1	106.38 (8)
O2^iii^—Th1—O6^v^	134.16 (6)	O3—V1—O1	110.92 (8)
O7^iv^—Th1—O6^v^	149.25 (5)	O2—V1—O4	110.99 (9)
O9—Th1—O6^v^	70.53 (6)	O3—V1—O4	111.03 (8)
O3^i^—Th1—O8	66.51 (6)	O1—V1—O4	106.04 (8)
O1^ii^—Th1—O8	66.54 (6)	O5—V2—O6	111.61 (9)
O2^iii^—Th1—O8	131.90 (7)	O5—V2—O7	112.45 (9)
O7^iv^—Th1—O8	75.03 (6)	O6—V2—O7	99.47 (8)
O9—Th1—O8	126.93 (8)	O5—V2—O4	110.14 (8)
O6^v^—Th1—O8	77.61 (6)	O6—V2—O4	110.71 (8)
O3^i^—Th1—O7	140.50 (5)	O7—V2—O4	112.08 (8)
O1^ii^—Th1—O7	73.42 (5)	V2—O4—V1	136.90 (10)
O2^iii^—Th1—O7	69.92 (5)	H8A—O8—H8B	104.5
O7^iv^—Th1—O7	66.76 (6)	H9A—O9—H9B	104.5
O9—Th1—O7	104.44 (8)		

Symmetry codes: (i) *x*−1, *y*, *z*+1; (ii) *x*, *y*, *z*+1; (iii) −*x*+1, −*y*+1, −*z*+1; (iv) −*x*+1, −*y*+1, −*z*+2; (v) −*x*+1, −*y*, −*z*+2.

### Hydrogen-bond geometry (Å, °)

**Table:**

*D*—H···*A*	*D*—H	H···*A*	*D*···*A*	*D*—H···*A*
O8—H8A···O4^ii^	0.86	2.47	3.114 (3)	133
O8—H8B···O5^i^	0.86	1.92	2.779 (3)	175
O9—H9A···O1^vi^	0.86	1.77	2.594 (3)	160
O9—H9B···O5^vii^	0.86	1.90	2.741 (3)	166

Symmetry codes: (ii) *x*, *y*, *z*+1; (i) *x*−1, *y*, *z*+1; (vi) −*x*+1, −*y*, −*z*+1; (vii) *x*−1, *y*, *z*.
